# 1-year prospective real life monitoring of asthma control and quality of life in Italy

**DOI:** 10.1186/1465-9921-13-112

**Published:** 2012-12-06

**Authors:** Claudio Terzano, Giovanni Cremonesi, Giuseppe Girbino, Eleonora Ingrassia, Serafino Marsico, Gabriele Nicolini, Luigi Allegra

**Affiliations:** 1 Respiratory Diseases Unit, Department of Cardiovascular and Respiratory Sciences, ‘Sapienza’ University of Rome, Viale del Policlinico 155, 00161, Rome, Italy; 2 Medical Affairs Department, Chiesi Farmaceutici S.p.A, Via Palermo 26/A, 43122, Parma, Italy; 3 Institute of Respiratory Medicine, University of Messina, Via Consolare Valeri, Gazzi, 98125, Messina, Italy; 4 Department of Respiratory Medicine, Azienda Ospedaliera Monaldi, Second University of Naples, Via Leonardo Bianchi, 80131, Naples, Italy; 5 Thoracopulmonary Department, University of Milan, Fondazione IRCCS Cá Granda Ospedale Maggiore Policlinico, Via Francesco Sforza 28, 20122, Milan, Italy

## Abstract

**Objectives:**

The study aimed at prospectively evaluating the evolution of asthma control in Italy, to evaluate the reasons for lack of asthma control, perceived quality of life (QoL) and association with level of asthma control, the impact of pharmacological treatment, the number of exacerbations and the healthcare resource consumption.

**Methods:**

PRISMA (PRospectIve Study on asthMA control) was an observational study performed in asthmatic patients including a cross-sectional phase and a 12-month prospective phase. Asthma control was assessed with the Asthma Control Test™ (ACT) and QoL was evaluated with EuroQoL-5D questionnaire filled in and collected during 5 clinic visits together with all the other data.

**Results:**

The prospective phase included 1017 patients with uncontrolled (55.7%) or partly controlled asthma (44.3%). Out of the 739 patients evaluable after 12 months, 22.2% achieved full asthma control (ACT score = 25) and 58.7% reached a good control (ACT score: 20–24). The improvement in asthma control was associated with improved QoL and reduced hospital visits. The main reasons for lack of asthma control were comorbidities, continued exposure to irritants/triggers and poor adherence to therapy. The frequency of exacerbations was lower in patients with controlled asthma.

A fixed combination therapy with an inhaled corticosteroid and a long-acting β2 agonist was reported by 77.0% of patients. A better asthma control and improved QoL were achieved with extrafine beclomethasone/formoterol compared to either budesonide/formoterol or fluticasone/salmeterol.

**Conclusions:**

An improvement in asthma control and QoL can be achieved during a 1-year monitoring in a real life setting. Extrafine beclomethasone/formoterol was associated with significant benefit in terms of asthma control and QoL compared to large-particles combinations.

ClinicalTrials.gov number NCT01110460.

## Introduction

According to international guidelines, once treatment has been established, therapeutic management of asthma should be based on asthma control, rather than asthma severity [1,2]. Patients with controlled asthma can prevent the majority of attacks, avoid daytime and nighttime symptoms, stay physically active and have reduced risk to exacerbate [3].

Recognizing the importance of the patient’s perspective and of the poor correlation between lung function and symptoms [3,4], clinical trials and clinical practice have increasingly focused on the assessment of asthma control. This is a general term that implies a global assessment of actual status and future risk including symptoms, reliever use, lung function, and the frequency/severity of exacerbations [5]. The level of asthma control is usually categorized into controlled, partly controlled and uncontrolled [1]. Moreover, a positive correlation between asthma control and quality of life (QoL) has been demonstrated [3]. Awareness of patient reported outcomes such as asthma control and QoL is increasing, together with an emphasis in clinical research and by regulatory bodies because of their relevance in the overall treatment efficacy assessment [5].

The PRISMA (PRospectIve Study on asthMA control) observational study was designed to include a cross-sectional phase and a 12-month prospective phase in order to estimate the level of asthma control in real life and its evolution during a 1-year follow up. The results of the cross-sectional phase of the PRISMA study, investigating the level of asthma control in 2853 patients with asthma recruited in 56 respiratory clinics in Italy have been recently published [6]. The main findings indicate that despite a high proportion of patients have controlled asthma, one third of patients are still uncontrolled or partly controlled. Previous studies were available evaluating the control of asthma but comprised smaller populations and with a less representative distribution throughout the country [7-11]. In the PRISMA study, patients filled in validated questionnaires for asthma control and QoL measurement and data were collected during visits by respiratory specialists. In contrast, previous studies have collected data by telephone interviews [7-9], web-based questionnaires [11-13], or postal screening questionnaires [14].

Moreover, all the above studies are cross-sectional but only prospective monitoring of patients’ asthma control using composite measures can describe how this reflect asthma outcomes and/or future risks [5]. Unfortunately, no information is yet available on the rate of achievement of asthma control among poorly controlled asthma patients. It has been suggested that monitoring outcomes and taking appropriate action through regular visits may improve current levels of asthma control [15]. Behavioral factors such as smoking and non-adherence may reduce the efficacy of treatment and patients' perceptions influence these behaviors. Under-treatment may also be related to patients' underestimation of the significance of symptoms, and lack of awareness of achievable control. There is a need to raise patient expectations by increasing awareness of the QoL that could be attained.

The aim of the present study was to measure asthma control in a prospective 12 months observation period in patients whose asthma was classified as uncontrolled or partly controlled in a cross-sectional phase visit [6]. The prospective phase described in this manuscript aimed to evaluate the proportion of patients with uncontrolled or partly controlled asthma who achieve asthma control at 12 months. Secondary objectives were: to evaluate the reasons for lack of asthma control, perceived QoL and association with level of asthma control, the impact of pharmacological treatment, the number of exacerbations and the healthcare resource consumption.

## Methods

### Study design

The PRISMA study was designed to include a cross-sectional phase [6] and a 12-month prospective phase in order to estimate the level of asthma control in real life and its evolution during a 1-year follow up. Clinic visits were performed every 3 months for a total of 5 visits throughout the 1-year study period and data were collected at each visit. The results of the cross-sectional phase of the PRISMA study were previously published [6]. Patients with uncontrolled or partly controlled asthma in the cross-sectional phase were included in the longitudinal phase of the study described hereafter. According to the observational study design, no randomisation procedure was implemented during the enrolment. The assignment of the patient to a particular therapeutic strategy was not defined in the study protocol but fell within current practice according to the physician’s decision.

The protocol was approved by the Institutional Review Board of each centre, and informed written consent was obtained from each participant prior to study initiation.

### Patients

The first fifty consecutive patients visiting 56 Italian pneumology centers were included in the cross-sectional phase of the PRISMA study if they satisfied the following criteria: i) provided written informed consent; ii) adult patients, smokers and non-smokers, with a previous asthma diagnosis of at least six months; iii) patients able to understand and fill in the questionnaires. Exclusion criteria were: i) patients included in clinical trials or who have been attending one in the last 12 weeks; ii) patients with severe and disabling diseases; iii) pregnant women.

Only patients who had uncontrolled or partly controlled asthma according to the ACT score in the cross-sectional visit were included in the prospective phase here described.

### Patient reported outcomes

The self-evaluating Asthma Control Test™ (ACT) was used to determine the patients' level of disease control in the 4 weeks preceding each clinic visit [4,16]. The overall ACT score varies from 5 (very poor asthma control) to 25 (full asthma control) with scores ranging from 20 to 24 defining controlled asthma, scores ranging from 16 to 19 partly controlled asthma and scores ≤15 uncontrolled asthma [2,4]. The ACT has been validated prospectively in several studies [5,17]. The EuroQoL-5D questionnaire [18] (EQ-5D) was used to determine the QoL perceived by the patient. It is a standardized self-rating test which refers to the day of rating applicable to a wide range of health conditions and treatments. EQ-5D provides a score where the maximum value of 1 denotes the best health status and 0 identifies a health status comparable to death. In addition, patients scaled their health on a visual analogue scale (VAS) ranging from 0 to 100, with 0 as the worst possible health status and 100 as the best possible health status [18]. Questionnaires were completed by each participant at each visit.

### Other variables

Current antiasthmatic therapies were recorded at each visit (in terms of active ingredient, dosage, duration and administration method). Patients were defined as treated with a specific drug if they had evidence of treatment for at least 5 consecutive days in the 3 months before the visit. For the comparison of asthma control level among treatments, only patients who had been on that therapy in the last 4 weeks were included in the analysis to match with ACT that evaluates asthma control in the last 4 weeks [6]. Possible reasons for poor control were described according to doctors’ opinion.

Exacerbations were registered along with the consumption of healthcare resources due to asthma (hospitalizations, emergency room visits, outpatients visits) at each clinic visit.

Exacerbations were defined as worsening of symptoms requiring any of the following: an increase in therapy or systemic corticosteroids, unscheduled specialist visit or an access to an emergency room.

### Statistical analysis

Due to the lack of prospective data on asthma control in observational studies, the sample size was estimated on the basis of the AIRE cross-sectional study [8]. The sample size was determined in order to have a relative error lower than 30% based on available literature data. It was estimated that a sample size of at least 1270 patients with uncontrolled or partly controlled asthma in the cross-sectional phase would have allowed to detect a rate of 5.1% of patients reaching optimal asthma control at the end of the 1-year prospective phase of the study, with a 95% CI equal to 5.1 ± 1.40% (estimating a 25% drop-out rate).

Summary statistics (mean, standard deviation) were provided for continuous variables, and absolute and relative frequency distribution was provided for categorical data. Comparisons between categorical variables were analyzed by the Chi-square test (or Fisher exact test, when appropriate). Comparisons between continuous variables were performed by the Kruskal-Wallis test. Post-hoc comparisons were performed, when applicable: in such cases, Bonferroni's correction was applied. Multivariate linear regression analyses were used in order to assess the association between the QoL (EQ-5D and VAS scores) and asthma control at the 12-month follow-up visit. Age, gender, educational level, BMI, smoking habits, asthma risk factors/triggers in the occupational environment, concomitant disease, QoL and asthma control (in the cross-sectional visit) were included as covariates. Since the independent variables have different scales, covariates were standardized. Significance was set at a two-tailed p-value of 0.05. All statistical analyses were performed by using the statistical analysis software "SAS version 9.2".

## Results

### Study population

**Figure 1:**
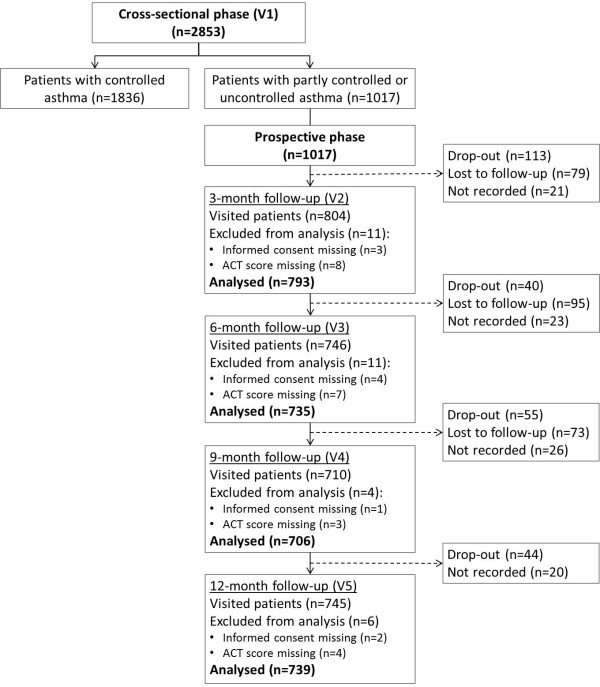
**Patient flow.** Patients were considered as drop-out if they definitely withdrew from the study; patients were considered as lost to follow-up if they missed one visit but they attended to a subsequent follow-up visit.

Clinical characteristics of patients with uncontrolled or partly controlled asthma in the cross-sectional phase are summarized in Table 1 and patient flow during the 12-month observation period is described in Figure 1. A total of 1017 uncontrolled or partly controlled patients were enrolled from January to October 2009 and followed up for 12 months with approximately 70% attending follow-up visits (Figure 1). The mean age was 46 years and 66.3% (n = 674) were females. Patients with uncontrolled asthma were 566 (55.7%), those with partly controlled asthma were 451 (44.3%). Comparing patient clinical characteristics between the uncontrolled and partly controlled groups, a few significant differences were observed: uncontrolled patients reported that they had a poorer patient-doctor communication compared to the partly controlled patients (25.3% vs. 16.2%, p < 0.001); the percentage of obese patients was higher (22.3% vs. 16.6%, p = 0.021) and sinusitis was more frequent among the uncontrolled patients (13.4% vs. 8.4%, p = 0.012).

**Table 1:** **Patients characteristics at the cross**-**sectional phase visit**

	**Total**	**Partly controlled**	**Uncontrolled**
Number of patients, n (%)	1017 (100)	451 (44.3)	566 (55.7)
Age, years, mean (SD)	46 (15)	45 (15)	47 (15)
Gender, n (%)			
Males	343 (33.7)	160 (35.5)	183 (32.3)
Females	674 (66.3)	291 (64.5)	383 (67.7)
Duration of asthma, years, mean (SD)	17.4 (13.0)	16.4 (12.7)	18.2 (13.2)
Body mass index categories, n (%)			
Obese (≥ 30 kg/m^2^) ^(1)^	201 (19.8)	75 (16.6)	126 (22.3)
Overweight (≥ 25 and < 30 kg/m^2^)	357 (35.1)	162 (35.9)	195 (34.5)
Normal weight (≥ 18.5 and < 25 kg/m^2^)	390 (38.4)	188 (41.7)	202 (35.7)
Underweight (< 18.5 kg/m^2^)	32 (3.2)	12 (2.7)	20 (3.5)
Not available	37 (3.6)	14 (3.1)	23 (4.1)
Smoking habits, n (%)			
Current smokers	197 (19.4)	77 (17.1)	120 (21.2)
Non-smokers	637 (62.6)	287 (63.6)	350 (61.8)
Ex-smokers ^(2)^	174 (17. 1)	86 (19.1)	88 (15.6)
Not available	9 (0.9)	1 (0.2)	8 (1.4)
Concomitant diseases ^(3)^, n (%)			
Total	640 (62.9)	287 (63.6)	353 (62.4)
Rhinitis	362 (35.6)	164 (36.4)	198 (35.0)
Cardiovascular diseases	151 (14.8)	70 (15.5)	81 (14.3)
Gastro-oesophageal reflux	171 (16.8)	68 (15.1)	103 (18.2)
Sinusitis ^(4)^	114 (11.2)	38 (8.4)	76 (13.4)
Nasal polyposis	52 (5.1)	25 (5.5)	27 (4.8)
Type 2 diabetes	39 (3.8)	14 (3.1)	25 (4.4)
Respiratory infections	43 (4.2)	15 (3.3)	28 (5.0)
Psychological disturbances	25 (2.5)	9 (2.0)	16 (2.8)
Type 1 diabetes	5 (0.5)	2 (0.4)	3 (0.5)
Other diseases	108 (10.6)	42 (9.3)	66 (11.7)
Quality of life, n	1010	448	562
EQ-5D ^(5)^ score, mean (SD)	0.72 (0.2)	0.75 (0.2)	0.69 (0.2)
EQ-5D ^(5)^ VAS score, mean (SD)	65.1 (15.6)	68.6 (14.9)	61.5 (16.3)

^(1)^ Overall p = 0.021; ^(2)^ Patients who stopped smoking since at least one year; ^(3)^ Patients could have more than one concomitant disease; ^(4)^ Overall p = 0.012; ^(5)^ EQ-5D: EuroQoL-5D questionnaire.

The most frequently reported therapies (at least 5 consecutive days of therapy in the last 3 months) were: a fixed combination of an inhaled corticosteroid and a long acting β2 agonist (ICS/LABA) in 48.3% of patients; leukotriene receptor antagonists in 23.6% and ICS in 11.2%.

Clinical characteristics of patients (n = 739) attending the 12-month follow-up visit are summarized in Table 2.

**Table 2:** **Patient characteristics at the 12**-**month follow**-**up visit**

	**Total**	**Controlled**	**Partly controlled**	**Uncontrolled**	**Overall p**-**value**
Number of patients, n (%)	739	598 (80.9)	87 (11.8)	54 (7.3)	
Smoking habits, n (%) ^(1)^					0.437
Current smokers	136 (18.4)	113 (18.9)	14 (16.1)	9 (16.7)	
Non-smokers	468 (63.3)	384 (64.2)	50 (57.5)	34 (63.0)	
Ex-smokers ^(2)^	128 (17.3)	97 (16.2)	21 (24.1)	10 (18.5)	
Not available	7 (0.9)	4 (0.7)	2 (2.3)	1 (1.9)	
Improvement of 3 points in ACT score, n (%)	642 (86.9)	572 (95.7)	55 (63.2)	15 (27.8)	<0.001^(3)^
Quality of life, n	733	593	86	54	
EQ-5D^(4)^ score, mean (SD)	0.87 (0.19)	0.91 (0.13)	0.71 (0.23)	0.62 (0.30)	<0.001^(5)^
EQ-5D^(4)^ VAS score, mean (SD)	80.0 (13.6)	83.8 (10.2)	67.2 (12.1)	58.9 (16.0)	<0.001^(6)^
Antiasthmatic therapy, n (%) ^(7)^					
No therapy	59 (8.0)	55 (9.2)	3 (3.5)	1 (1.9)	-
ICS /LABA	602 (81.5)	484 (80.9)	72 (82.8)	46 (85.2)	-
LTRA	237 (32.1)	165 (27.6)	42 (48.3)	30 (55.6)	-
ICS	71 (9.6)	55 (9.2)	7 (8.1)	9 (16.7)	-
Short-acting β_2_agonist	42 (5.7)	21 (3.5)	13 (14.9)	8 (14.8)	-

^(1)^ Smoking habits was reported only at the cross-sectional phase visit; ^(2)^ Patients who stopped smoking since at least one year; ^(3)^ Bonferroni’s corrected p < 0.001 for all comparisons; ^(4)^ EQ-5D: EuroQoL-5D questionnaire; ^(5)^ Bonferroni’s corrected p < 0.001 patients with controlled asthma vs. patients with partly controlled and uncontrolled asthma; Bonferroni’s corrected p = 0.060 patients with partly controlled asthma vs. patients with uncontrolled asthma; ^(6)^ Bonferroni’s corrected p < 0.001 patients with controlled asthma vs. patients with partly controlled and uncontrolled asthma; Bonferroni’s corrected p = 0.004 patients with partly controlled asthma vs. patients with uncontrolled asthma; ^(7)^ Data are percentage of patients in each category of asthma control reporting therapies for at least 5 consecutive days in the last 3 months; patients could have more than one pharmacologic class therapy; ICS: inhaled corticosteroids; LABA: long-acting β_2_ agonist; LTRA: leukotriene receptor antagonists.

### Asthma control

**Figure 2:**
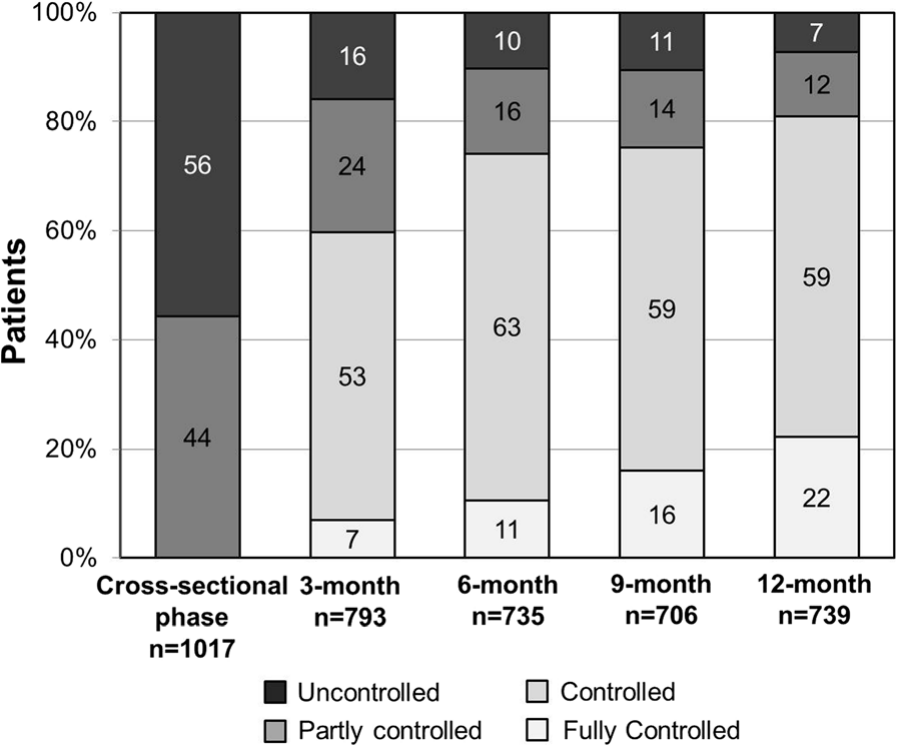
**Evolution of asthma control during the 12-month prospective phase.** Data are presented as percentage of patients in each category of asthma control: fully controlled (ACT score = 25), controlled (ACT score: 24–20), partly controlled (ACT score: 19–16) and uncontrolled (ACT score ≤ 15).

An improvement in asthma control was observed at the first follow-up visit after 3 months, where 6.9% of patients achieved full asthma control (Figure 2). Furthermore, the proportion of patients achieving full asthma control increased over time, reaching 22.2% on the last visit, with 58.7% of patients having controlled asthma, 11.8% partly controlled asthma and 7.3% were still uncontrolled.

The main reasons for lack of asthma control as declared by treating physicians were comorbidities in 36.2% of patients, continued exposure to irritants/triggers in 34.0%, and poor adherence to therapy in 27.0% (Table 3).

**Table 3:** **Comparison between the reasons for lack of asthma control at the cross**-**sectional visit and at the 12**-**month follow**-**up visit**

**Reasons for lack of asthma control**, **n(%)**^**(1)**^	**Cross**-**sectional phase visit**	**12**-**month follow**-**up visit**	
	**Total n = 1017**	**Total n = 141**	**p-value**^**(2)**^
Comorbidities	154 (15.1)	51 (36.2)	<0.001
Continued exposure to irritants/triggers	295 (29.0)	48 (34.0)	0.238
Poor adherence to therapy	440 (43.3)	38 (27.0)	<0.001
Smoking habits	154 (15.1)	11 (7.8)	0.020
Lack of patient follow up	405 (39.8)	9 (6.4)	<0.001
Inadequate therapy	202 (19.9)	8 (5.7)	<0.001
Poor patient-doctor communication	216 (21.2)	5 (3.6)	<0.001
Inadequate inhalation technique	63 (6.2)	4 (2.8)	0.125

^(1)^ According to the doctor’s opinion; Patients could have more than one reason; ^(2)^ Fisher exact test between the cross-sectional visit and the 12-month follow-up visit.

When comparing reasons for poor control after 1-year follow-up with those reported in the cross-sectional visit, poor adherence to therapy, lack of patient follow-up, poor patient-doctor communication and inadequate therapy were less frequently reported, whereas comorbidities were reported more frequently (Table 3).

Among patients treated with ICS/LABA combinations, in the last 4 weeks before the end of the study (n = 569), 19.0% had fully controlled asthma, 61.7% had controlled asthma, 12.0% had partly controlled asthma and 7.4% had uncontrolled asthma.

**Figure 3:**
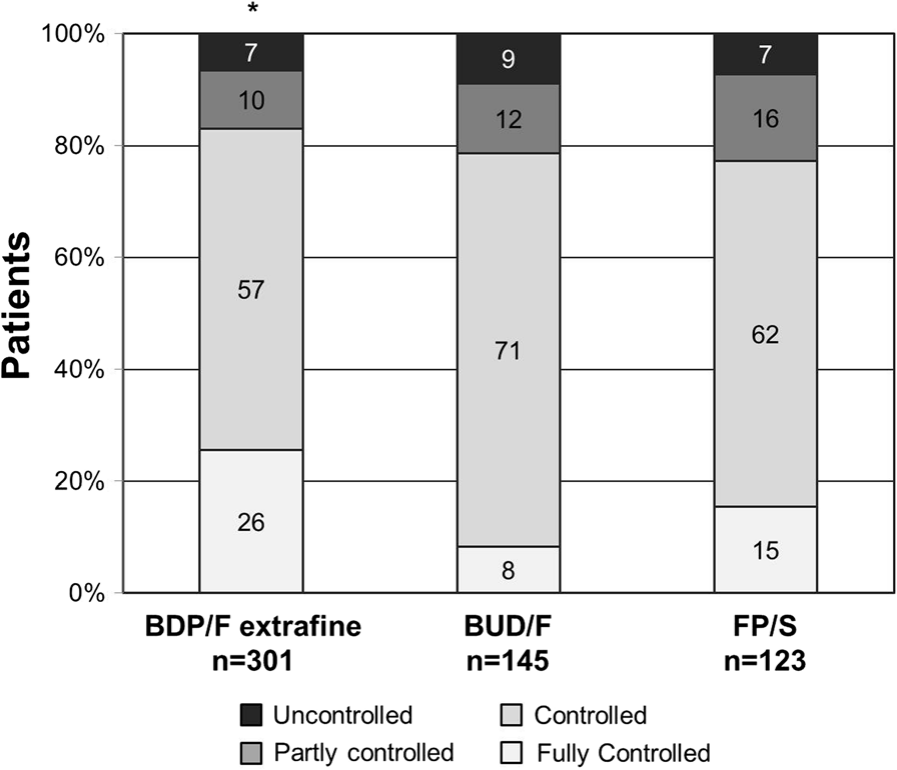
**Percentage of patients treated with different ICS/LABA fixed combinations with fully controlled (ACT score = 25), controlled (ACT score: 24–20), partly controlled (ACT score: 19–16) and uncontrolled (ACT score ≤ 15) asthma at the 12-month follow-up visit.** BDP/F = beclomethasone/formoterol; BUD/F = budesonide/formoterol; FP/S = fluticasone/salmeterol. *Bonferroni’s corrected p < 0.001 BDP/F vs. BUD/F (for fully controlled patients).

Three hundred and one patients were in treatment with the extrafine beclomethasone/formoterol (BDP/F) combination in a pressurized Metered Dose Inhaler (pMDI), 145 were in treatment with budesonide/formoterol (BUD/F) Dry Powder Inhaler (DPI) and 123 with fluticasone/salmeterol (FP/S; 46.3% pMDI and 53.7% DPI). When comparing the different available combinations, a greater proportion of patients treated with the extrafine BDP/F combination achieved asthma control compared to patients treated with either BUD/F or FP/S (Table 4, Figure 3). The proportion of patients with full asthma control in the extrafine BDP/F-treated group was significantly greater than in the BUD/F-treated group (Bonferroni’s corrected p < 0.001; Figure 3). Moreover, an improvement of 3 points in the ACT score was achieved by a significantly greater proportion of BDP/F treated patients as compared to BUD/F treated patients (Table 4). The probability of having full asthma control was higher in patients treated with extrafine BDP/F than in those receiving BUD/F (odds ratio 3.8; 95% adjusted CI: 1.733 to 8.374; Bonferroni’s corrected p < 0.001) and FP/S (odds ratio 1.9; 95% adjusted CI: 0.957 to 3.699; Bonferroni’s corrected p = 0.075).

**Table 4:** **Characteristics of patients treated with ICS**/**LABA fixed combinations for at least 4 weeks before the 12**-**month follow**-**up visit**

	**Total n** = **569**	**BDP**/**F extrafine n** = **301**	**BUD**/**F n** = **145**	**FP**/**S n** = **123**	**Overall p**-**value**
Level of asthma control, n (%)					0.001^(1)^
Fully controlled	108 (19.0)	77 (25.6)	12 (8.3)	19 (15.4)	
Controlled	351 (61.7)	173 (57.5)	102 (70.3)	76 (61.8)	
Partly controlled	68 (12.0)	31 (10.3)	18 (12.4)	19 (15.4)	
Uncontrolled	42 (7.4)	20 (6.6)	13 (9.0)	9 (7.3)	
Current smokers, n (%)	110 (19.3)	69 (22.9)	25 (17.2)	16 (13.0)	0.617
Fully controlled	25 (22.7)	20 (29.0)	3 (12.0)	2 (12.5)	0.135
Controlled	66 (60.0)	39 (56.5)	17 (68.0)	10 (62.5)	
Partly controlled	12 (10.9)	7 (10.1)	3 (12.0)	2 (12.5)	
Uncontrolled	7 (6.4)	3 (4.4)	2 (8.0)	2 (12.5)	
ACT score, mean (SD)	21.5 (3.4)	22.0 (3.4)	20.7 (3.6)	21.3 (3.3)	<0.001^(2)^
Improvement of 3 points in ACT score, n (%)	493 (86.6)	268 (89.0)	116 (80.0)	109 (88.6)	0.024^(3)^
Daily Dose of ICS, n	537	287	141	122	
Mean mcg (SD)	NA	318.8 (114.3)	651.1 (291.2)	750.6 (371.7)	<0.001^(4)^
Patients with exacerbation, n (%) ^(5)^	84 (14.8)	40 (13.3)	19 (13.1)	25 (20.3)	0.145

BDP/F: beclomethasone/formoterol; BUD/F: budesonide/formoterol; FP/S: fluticasone/salmeterol; ^(1)^ Bonferroni’s corrected p = 0.001 BDP/F vs. BUD/F; ^(2)^ Bonferroni’s corrected p < 0.001 BDP/F vs. BUD/F; Bonferroni’s corrected p = 0.039 BDP/F vs. FP/S; ^(3)^ Bonferroni’s corrected p = 0.029 BDP/F vs. BUD/F; ^(4)^ Bonferroni’s corrected p < 0.001 BDP/F vs. BUD/F and FP/S; Bonferroni’s corrected p < 0.001 BUD/F vs. FP/S; ^(5)^ Reported in the last 3 months before the 12-month visit.

The mean daily dose of ICS administered was approximately 2-fold lower for extrafine BDP/F compared to either BUD/F or FP/S (Table 4).

### Quality of life and exacerbations

The EQ-5D and VAS scores at the 12-month follow-up visit were higher in patients with controlled asthma as compared to patients with partly controlled or uncontrolled asthma (Table 2). When covariates were taken into account, control at the 12-month visit, age and QoL in the cross-sectional visit were shown to be associated with 12-month QoL (p < 0.001).

**Figure 4:**
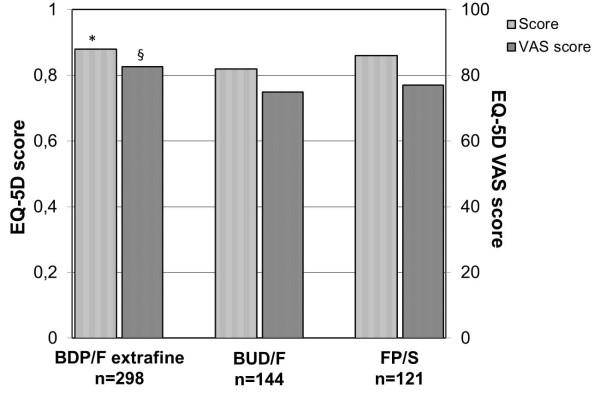
**Quality of life (EQ-5D score and VAS) in patients treated with different ICS/LABA fixed combinations at the 12-month follow-up visit.** BDP/F = beclomethasone/formoterol; BUD/F = budesonide/formoterol; FP/S = fluticasone/salmeterol. Data are means. *Bonferroni’s corrected p = 0.001 BDP/F vs. BUD/F; ^§^Bonferroni’s corrected p < 0.001 BDP/F vs. BUD/F and FP/S.

Patients treated with extrafine BDP/F had a significantly higher EQ-5D score compared to BUD/F (0.88 ± 0.18 vs. 0.82 ± 0.19, Bonferroni’s corrected p = 0.001; Figure 4). Furthermore, a significantly higher VAS score was detected in the extrafine BDP/F-treated group (82.7 ± 12.5) compared to either BUD/F (74.9 ± 14.1, Bonferroni’s corrected p < 0.0001) or FP/S-treated patients (77.0 ± 13.6, Bonferroni’s corrected p < 0.001; Figure 4).

A total of 589 exacerbations were reported during the 1-year follow up. The percentage of patients reporting exacerbations was lower, at all follow-up visits, in patients with controlled asthma compared to the sum of patients with partly controlled and uncontrolled asthma (p < 0.001). In the last 3 months before the end of the study, at least one exacerbation was reported by 67% of patients with partly controlled and uncontrolled asthma compared to 6% of patients with controlled asthma (p < 0.001).

The percentage of patients reporting exacerbations in the last 3 months before the 12-month visit was 13.3% in patients treated with extrafine BDP/F, 13.1% in patients treated with BUD/F and 20.3% in patients treated with FP/S.

### Consumption of healthcare resources

**Figure 5:**
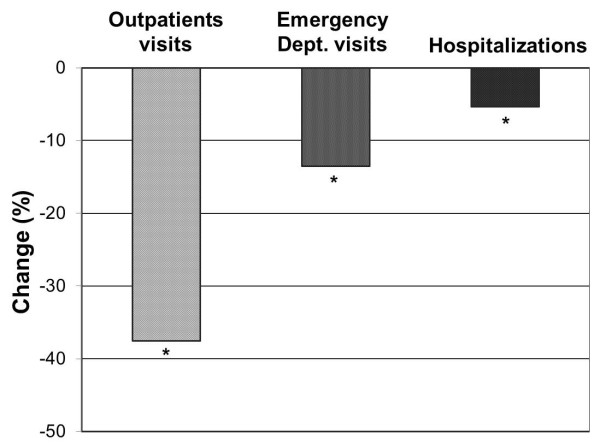
**Percentage change in healthcare resource consumption due to asthma from 3 months before the cross-sectional phase visit to 3 months before the 12-month follow-up visit.** *p < 0.001 for all comparisons.

A significant decrease in healthcare resource consumption was detected during the 1-year follow-up (Figure 5). The percentage of patients requiring an outpatient visit in the last 3 months before the 12-month visit was reduced approximately 3-fold to 24.5% (compared to 62% at the beginning of the study, p < 0.001). The proportion of patients that were admitted to hospital was also reduced from 5.8% in the cross-sectional visit to 0.5% in the 12-month follow-up visit (p < 0.001). The frequency of emergency room visits was also significantly reduced, from 15.4% in the cross-sectional visit to 1.9% in the last follow-up visit (p < 0.001).

## Discussion

PRISMA is the first prospective observational study specifically designed to evaluate the proportion of patients achieving asthma control during a 1-year follow-up in real life conditions.

The main findings from this prospective study demonstrate that 22.2% of patients that previously had partly controlled or uncontrolled asthma (n = 739) achieved full asthma control after 1 year, 58.7% had controlled asthma, 11.8% had partly controlled asthma and 7.3% were still uncontrolled.

Among those patients who, after the 1-year follow-up, did not attain asthma control (n = 141), the reasons were anyway different, according to the physician assessment, from those at study start. Actually, when comparing the last visit of the prospective phase with the cross-sectional visit, reasons for lack of asthma control changed from lack of adequate therapy/follow-up/communication or smoking habits, i.e. all external factors that therefore can be improved by a better asthma care, to the presence of comorbidities. This finding suggests that during 1-year monitoring in real life conditions, an improvement in patient behavioral factors that contribute to the lack of asthma control such as adherence to medication, patient-doctor communication and follow up can be obtained. An additional observation coming from the present study is that the rate of outpatients visits, hospitalizations and emergency room visits was reduced approximately 3-fold by the end of the follow-up period. This is in line with the AIRE study that examined the use of healthcare resources in 7 European countries and showed that worse asthma control was associated with an increased requirement for unscheduled care and higher costs [8].

Moreover, our findings indicate that asthma control can be achieved in a great proportion of patients treated with the ICS/LABA fixed combinations, which is in agreement with recent observational studies reporting the ICS/LABA combination being the most effective treatment choice [19-21]. In a 12-month office-based observational study, patients recently started on FP/S combination, had significantly greater improvement in both asthma control and QoL, compared with patients newly started on montelukast [20]. Another study with a 12-month real life observation period showed a greater change in asthma control score in FP/S treated patients compared to other forms of treatment that were in accordance with asthma treatment guidelines (including BUD/F but not including BDP/F extrafine) [21]. We observed that a greater proportion of patients treated with extrafine BDP/F fixed combination achieved good asthma control and the maximum ACT score (i.e. full control) compared to either BUD/F or FP/S -treated patients, the difference being significant versus BUD/F. Moreover, the proportion of patients achieving a clinically meaningful change in ACT score (3 points) was greater in BDP/F treated patients compared to BUD/F treated patients. We considered only patients who had been on the same therapy in the last 4 weeks for the comparison of asthma control level among treatments to match with ACT that evaluates asthma control in the last 4 weeks.

As regards QoL assessed by EQ-5D, an improvement of approximately 21% was observed after 1 year in the whole population studied. When looking at patients treated with ICS/LABA fixed combinations, extrafine BDP/F-treated patients experienced significantly higher QoL compared to either BUD/F or FP/S-treated patients at the end of the 12-month observation period. These results are consistent with those obtained in the cross-sectional phase of the PRISMA study [6] and may be attributed to the formulation of this combination, which is characterized by extrafine particles able to reach and treat the whole bronchial tree, including small airways [22]. Indeed, asthma control and QoL correlate with functional parameters reflecting small airways function and improvements in ventilation heterogeneity are associated with improvements in symptoms [23,24].

A better asthma control with extrafine BDP/F combination compared to BUD/F and FP/S combinations was recently shown in two observational cross-sectional studies [6,25]. This is also confirmed by results from a recent observational study which showed that initiating or increasing therapy with an extrafine beclomethasone formulation results in similar or better asthma control compared with non-extrafine fluticasone [26].

The evidence of improvement in asthma control and QoL in patients treated with extrafine formulations compared to large particles formulations is supported by randomized controlled trials (RCTs) [27,28]. Moreover, an RCT demonstrated that a greater asthma control was achieved in patients treated with extrafine BDP/F combination compared to the same drugs administered with separate inhalers delivering large particles [29]. This evidence is particularly interesting when compared with similarly designed studies with the other two available combinations, as shown in a recently published review, thus supporting the concept of greater efficacy of BDP/F due to extrafine particles [30].

The difference in asthma control was detected despite the lower ICS dose and the higher percentage of smokers in the BDP/F patient cohort. This is of particular interest since smoking is associated with an increased risk of poor control [31,32] and asthmatic smokers are less responsive to ICS therapy [33,34]. Our findings support the concept that the tobacco smoke-drug particle interactions, which contribute to drug resistance, are less likely to occur in case of extra-fine formulations [35]. Together, these results suggest that asthmatic smokers can particularly benefit from extrafine BDP/F combination. This is also supported by a recently published prospective real-life study showing significant improvements in pulmonary function and asthma control in patients treated with extrafine BDP/F combination with the same treatment benefits observed in former or current smokers compared with non-smoking asthmatics [36].

Someone may argue that the level of asthma control detected in the present study is quite high compared to the GOAL study in which patients were stepped up for a 1-year period to the highest recommended dose of FP/S combination [37]. However, this difference can be explained by the different patient population, definition of control and way of measuring it. In the GOAL study only patients with uncontrolled asthma were recruited and the definition of control as from asthma international guidelines included lung function. Moreover, the definition of “total control” used in the GOAL study was very stringent (defined by achievement of all specified criteria for 7 weeks in each 8-week period) and now abandoned [1,2]. By contrast, the ACT used in the present study is a validated patient reported outcome [4,5,16,17] and does not include lung function which has been shown having minimal impact on the reliability, responsiveness, and both cross-sectional and prospective validity of the instrument [38].

Observational studies present some limitations such as the possibility of unmeasured or unrecognized confounding factors or influences on prescribing. These results should be interpreted in light of the inherent limitations of non-randomized, uncontrolled studies. However, these limits are balanced by the broader applicability of observational real life data on larger and more representative patient populations with common co-morbidities and that can identify clinically important differences among treatments [39]. By contrast, RCTs have limited external validity as they have been performed on highly selected patient populations and most of the participants currently under asthma treatment in the community are not represented [40].

In conclusion, the main findings from this prospective phase of the PRISMA study demonstrate that an improvement in asthma control can be achieved during a 1-year monitoring in a real life setting. Furthermore, patients have improved QoL and reduced hospital visits. Fixed combination of extrafine BDP/F was associated with significant benefit in terms of asthma control and QoL compared to large-particles combinations, this advantage likely being attributed to the extrafine formulation.

## Competing interest

Giovanni Cremonesi: Employee of Chiesi Farmaceutici S.p.A, Eleonora Ingrassia: Employee of Chiesi Farmaceutici S.p.A, Gabriele Nicolini: Employee of Chiesi Farmaceutici S.p.A, Luigi Allegra: Consultant fees: GSK (I), Astrazeneca (I), Chiesi (I), Boehringer Mannheim (I), Zambon (I), IBSA (CH), Procter & Gamble (UK), Cotherix (USA). Reimbursements for attending a symposia: GSK (I), Astrazeneca (I), Chiesi (I), Boehringer Ingelheim (I), Eurodrug NL) Angelini (I). Fees for speaking: Menarini (I), Boehringer Ingelheim (I), IBSA (CH), Eurodrug (NL). Funds for research: Angelini (I). Funds for members of staff: Cotherix (USA) Exalee (USA), IBSA (CH). All other authors declare no competing interest.

## Authors' contributions

All of the authors contributed to the definition of the study protocol, data interpretation and writing of the manuscript. All of the authors read and approved the final manuscript
